# Familial chromosomal translocation X; 22 associated with infertility and recurrent X mosaicism

**DOI:** 10.1186/s13039-016-0249-5

**Published:** 2016-06-15

**Authors:** Juliana Dourado Grzesiuk, Ciro Silveira Pereira, Carlos Henrique Paiva Grangeiro, Clarissa Gondim Picanço-Albuquerque, Flávia Gaona Oliveira-Gennaro, Filipe Brum Machado, Enrique Medina-Acosta, Ester Silveira Ramos, Maisa Yoshimoto, Lucia Martelli

**Affiliations:** Genetics Department, Ribeirão Preto Medical School, University of Sao Paulo, Ribeirao Preto, 14049-900 Brazil; Center of Biotecnology and Cellular Therapy, San Raphael Hospital, Salvador, 41253-190 Brazil; Center of Biosciences and Biotechnology, Darcy Ribeiro State University of Northern of Rio de Janeiro, Campos dos Goytacazes, 28013-600 Brazil; Department of Medical Genetics, Faculty of Medical and Dentistry, University of Alberta, Edmonton, Canada

**Keywords:** Infertility, X; autosome translocation, recurrent mosaicism, X inactivation, array-CGH

## Abstract

**Background:**

Individuals with apparently balanced translocations, often, show no clinical findings. However, in meiosis, translocations tend to cause errors on chromosome disjunction and the ones involving sex chromosomes have particular implications for the phenotype. Male carriers of balanced X-autosome translocations are almost invariably infertile due to interruption of the spermatogenesis, but the mechanism is not fully understood.

**Case presentation:**

In this case report, we performed a combination of classical cytogenetics (G-banding), molecular cytogenetics (fluorescence in situ hybridization and X-chromosome inactivation study), and cytogenomics (microarray-based comparative genomic hybridization) techniques for characterization of an inherited (X;22) translocation in a family originally referred for infertility investigation. Both proband and his sister are infertile and present the maternally inherited translocation. Interestingly, the maternal grandmother was mosaic for X chromosome monosomy suggesting that the t(X;22) in the proband’s mother arose by errors at oogenesis. The presence of the same mosaicism of the X chromosome in the proband’s aunt is consistent with this consideration. Array- CGH analysis showed no constitutional pathogenic gains or losses in the translocation carriers. The X-chromosome inactivation studies revealed that the translocated X;22 was active in 99.3% of cells in the mother and in 88% of cells in the daughter. We suggest that incomplete skewing of X inactivation (>97 %) of the daughter could justify the infertility. This study is the first description of recurrent mosaicism of the X chromosome associated with a familial X-autosome translocation.

**Conclusions:**

The phenotype of infertility was probably caused by disruption of spermatogenesis due to gametogenesis specific errors resulted from meiotic pairing and segregation anomalies on the translocated chromosomes.

## Background

Reciprocal translocations are the most common balanced chromosomal rearrangements in humans [1]. Individuals with balanced translocations often present with no obvious phenotypic abnormalities, but may have a history of infertility. This phenotype occurs because chromosome disjunction and pairing between the translocated chromosomes may not normally occur at meiosis [2]. Such segregation anomalies during gametogenesis may result in infertility, an increased risk of spontaneous miscarriages or an abnormal phenotype in the offspring. The variable consequences depend on the structural constitution of the translocated chromosome, the length of the chromosomal region involved, the number and position of the breakpoints and their impact on chiasmata. Breakpoint regions in apparently balanced translocations seem to be more complex than previously considered and small genomic imbalances have been detected even in carriers with a normal phenotype [3, 4]. Translocations involving sex chromosomes have particular implications [5], and their impact in the male fertility is well documented in the literature [6].

Male carriers of balanced X-autosome translocations are almost invariably infertile due to interruption of the spermatogenesis, characterized by azoospermia or severe oligozoospermia [7]. The causes of this type of spermatogenesis failure are not fully understood. However, spermatogenesis is generally much more likely to involve meiotic disruption, when compared with oogenesis, due to the more efficient meiotic cell cycle checkpoints in male gametogenesis. This protection mechanism ensures that most potentially aneuploid gametes undergo cell death due to apoptosis. The effect of any disruption of male meiosis is thus likely to lead to a lowered sperm count [8]. Chromosomal translocations create specific pairing problems during meiotic recombination that often generates double strand DNA breaks. The presence of unrepaired DNA breaks can lead to aneuploid gametes since translocated autosomes which fail to synapse effectively can retain numerous unrepaired double-strand DNA breaks that may lead to meiotic arrest and impaired fertility. Unsynapsed chromosomes can be silenced during the meiotic phase of spermatogenesis, and this type of epigenetic regulation may also contribute to the meiotic arrest by silencing ‘meiosis-critical’ genes [9].

*De novo* balanced X-autosome translocations are considered to arise by errors in spermatogenesis [10]. This can be explained by the mispairing between the sex chromosomes during meiosis since the pairing only occurs on a short homologous region. Wrong pairing may allow crossing between heterologous chromosomes, resulting in translocation of chromosome segments. Although the origin of X-autosome translocations is usually paternal, its transmission happens mainly through the mother since male carriers are frequently infertile.

The correlation between the genotype and the phenotype of the patients can be established using classical and molecular cytogenetics techniques. The study of the genetic causes of infertility allows us to indicate the best treatment through assisted reproduction techniques and is important for the development of new approaches in the field of male infertility. Therefore, in this case report, we describe a unique (X;22) familial translocation associated with the infertility phenotype for a karyotype-phenotype correlation, using different methodologies.

## Case presentation

**Fig. 1:**
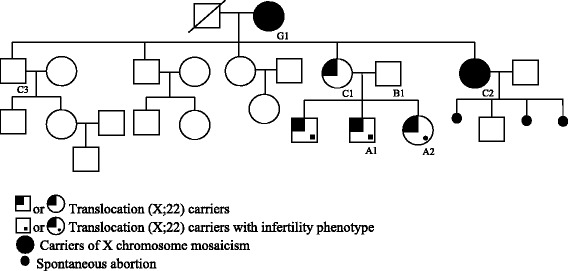
Family pedigree showing the carriers of the (X;22) translocation and X mosaicism

The proband (A1) is a married male, 30-years-old, carrier of a maternal translocation t (X;22). The couple did not use any methods of contraception for three years, without pregnancy. Physical examination showed diminished testis with parenchymatous consistency, normal epididymis and palpable deferens. Hormonal tests (FSH, LH, testosterone) were normal. Spermogram showed azoospermia. Additionally, the testicular biopsy showed hyalinization in the basal membrane of the seminiferous tubule and germinative cells in the initial stages of maturation. His youngest sister (A2) is 27 years old, married with no children, carrying the same apparently balanced translocation t (X;22). She is phenotypically normal and was referred to the Human Reproduction Division due to infertility. She is attempting pregnancy and has been under ovarian stimulation treatment. Their mother (C1) had three children and all carried the translocation t (X;22). The proband’s father (B1) present both normal phenotype and karyotype. The maternal aunt (C2) had three spontaneous abortions and one normal son. The maternal uncle (C3) had two children and one grandson, all of them with normal phenotype. The maternal grandmother (G1) gave birth to two healthy sons and three healthy daughters. Figure 1 shows the family pedigree.

Peripheral blood samples were collected from the patient and all cited relatives (A2, B1, C1, C2, C3, G1). Metaphase chromosomes were prepared using standard cytogenetic methods and 100 metaphases were analyzed by G-banding. Both proband and sister (A1, A2) had an apparently balanced translocation between chromosomes X and 22, of maternal origin, classified as: t (X;22) (22qter → 22q11.2::Xp22.3 → Xqter;22pter → 22q11.2:: Xp22.3 → Xpter). Their father (B1) and uncle (C3) presented normal karyotypes. Cytogenetic analysis of their aunt (C2) and grandmother (G1) identified a low level mosaicism for chromosome X aneuploidy. The karyotype of the aunt was: 45, X [09]/47, XXX [01]/46, XX [90] and of the grandmother was 45, X [13]/47, XXX [05]/48, XXXX [01]/46, XX [81]. None of them has any phenotypic features consistent with Turner syndrome.

### Molecular cytogenetics studies

The fluorescence in situ hybridization (FISH) technique was performed on metaphase chromosomes of the translocated patients by standard procedures, using Whole Chromosome Painting (WCP) probes for chromosomes X (LPP 0XR, Cytocell, UK) and 22 (LPP 22G, Cytocell, UK). FISH analysis showed a fragment of chromosome 22 translocated to chromosome X but we were unable to detect any segment of the X chromosome translocated to chromosome 22 likely because the reciprocal region involved on the X chromosome was too small to generate signal using a conventional paint probe.

**Fig. 2:**
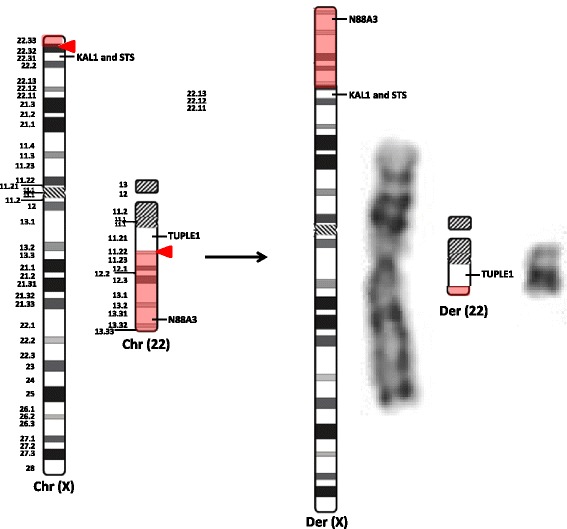
FISH and Cytogenetic analyses of the translocation cariers. **a**) FISH technique from patient A2 showing the subtelomeric probe of chromosome 22 (in green) in the distal portion of the short arm of the translocated X chromosome, while probe for TUPLE1 gene is located at band q11.21 (in red), remaining on the der(22). **b**) On the left side the ideograms from normal chromosomes X and 22 show the breakpoints (red arrow) and the translocated segments (shaded in red). On the right side are the schematic ideograms and the GTG banding pattern of the derivative chromosomes

FISH analysis using the probes LSI DiGeorge/VCFS TUPLE 1 (LPU 004, Cytocell, UK) and Kallmann/Steroid Sulphatase (LPU 016, Cytocell, UK) has showed that both genes *STS* and *KAL1* (also known as *ANOS1*) mapped to p22.31 remained on the derivative X chromosome and did not translocate to the chromosome 22. Additionally, the subtelomeric probe of chromosome 22 (N85A3) was displayed in the terminal portion of the short arm of the translocated X chromosome, while the *TUPLE1* gene (also known as *HIRA*), mapped to 22q11.21, remained on the chromosome 22 translocated (Fig. 2a). Figure 2b shows a schematic image of the position of the genes investigated by FISH in normal and translocated chromosomes. These results suggest that the translocation may involve the distal bands of the X chromosome, as p22.32 or p22.33 and that the breakpoint on chromosome 22 occurred between q11.21 and the subtelomeric region. Thus the definitive karyotype was: t (X;22) (22qter → 22q11.2::Xp22.3 → Xqter;22pter → 22q11.2::Xp22.3 → Xpter) [20].ish t (X;22) (N85A3+, wcpX+, wcp22+ KAL1+, STS+; wcpX-, wcp22+, TUPLE1+) [10].

### Genomic investigation

Genomic DNA was isolated from peripheral blood from the proband and his six mentioned relatives, using QIAamp DNA Blood Mini Kit (Qiagen, The Netherlands) and the microarray-based comparative genomic hybridisation (array-CGH) was performed using the platform Human Genome CGH Microarray Chip Kit, 2x400K (Agilent, USA) following the manufacturer’s protocol. This platform has 420,288 probes with an average spacing of 5.3 kb. In brief, genomic DNA from peripheral blood of each patient was fragmented and labeled with Cy3-dCTP or Cy5-dCTP using a Genomic DNA Enzymatic Labeling Kit (Agilent, USA). Samples were then hybridized on arrays for 40 h at 65 °C. After washing, the samples were scanned using the DNA microarray scanner SureScan (Agilent). The scan images were analyzed using the feature extraction software v10.5 (Agilent) and further analyzed with Nexus6.0. Copy number variations (CNVs) were taken into consideration when four or more flanking probes exceeded a value of the intensity ratios four times the standard deviation of the log 2 of all intensity ratios for that experiment. CNVs were considered as pathogenic or benign as suggested by the literature [11].

No significant copy gains and losses were detected by array-CGH analysis in the patients or their relatives in the genomic profiles of the chromosomes involved in the translocation or any other chromosome.

### X-chromosome inactivation assay

X-chromosome inactivation (XCI) was investigated by a methylation-sensitive restriction enzyme-based PCR indirect assay [12]. Briefly, 200 ng of genomic DNA were digested with *Hpa*II (Invitrogen, Carlsbad, CA, USA), for 2 h at 37 °C, or mock-digested. DNA genotyping was carried out in quantitative fluorescence polymerase chain biplex reactions (QF-PCR) in approximately 20 ng of digested or mock-digested DNA using 0.8 μM (*AR*) and 1.2 μM (*RP2*) of each primer pair. The forward primers were labeled at the 5′ end with the fluorochrome 6-FAM. The allele profiles were determined in an ABI 310 Prism Genetic Analyzer (Applied Biosystems). The data were analyzed with GeneScan Analysis 3.7 and Genotyper 3.7 software (Applied Biosystems). The proportion of blood cells carrying the active or the inactive X was estimated as described by Busque et al. [13].

This assay is based on methylation statuses of CpG near the short tandem repeats from the promoter region of retinitis pigmentosa 2 (*RP2*) gene and exon 1 of androgen receptor (*AR*) gene on X-chromosome. Cleavage with the restriction enzyme *Hpa*II is blocked when the DNA is methylated. After digestion, only uncleaved alleles are amplified by the QF-PCR. The ratio between the area under allele peaks after *Hpa*II cleavage was used to estimate the proportion of inactivation of each X chromosome. The size of the area under the informative allelic peak is inversely proportional to the percentage of cells with the corresponding active X chromosome.

**Fig. 3:**
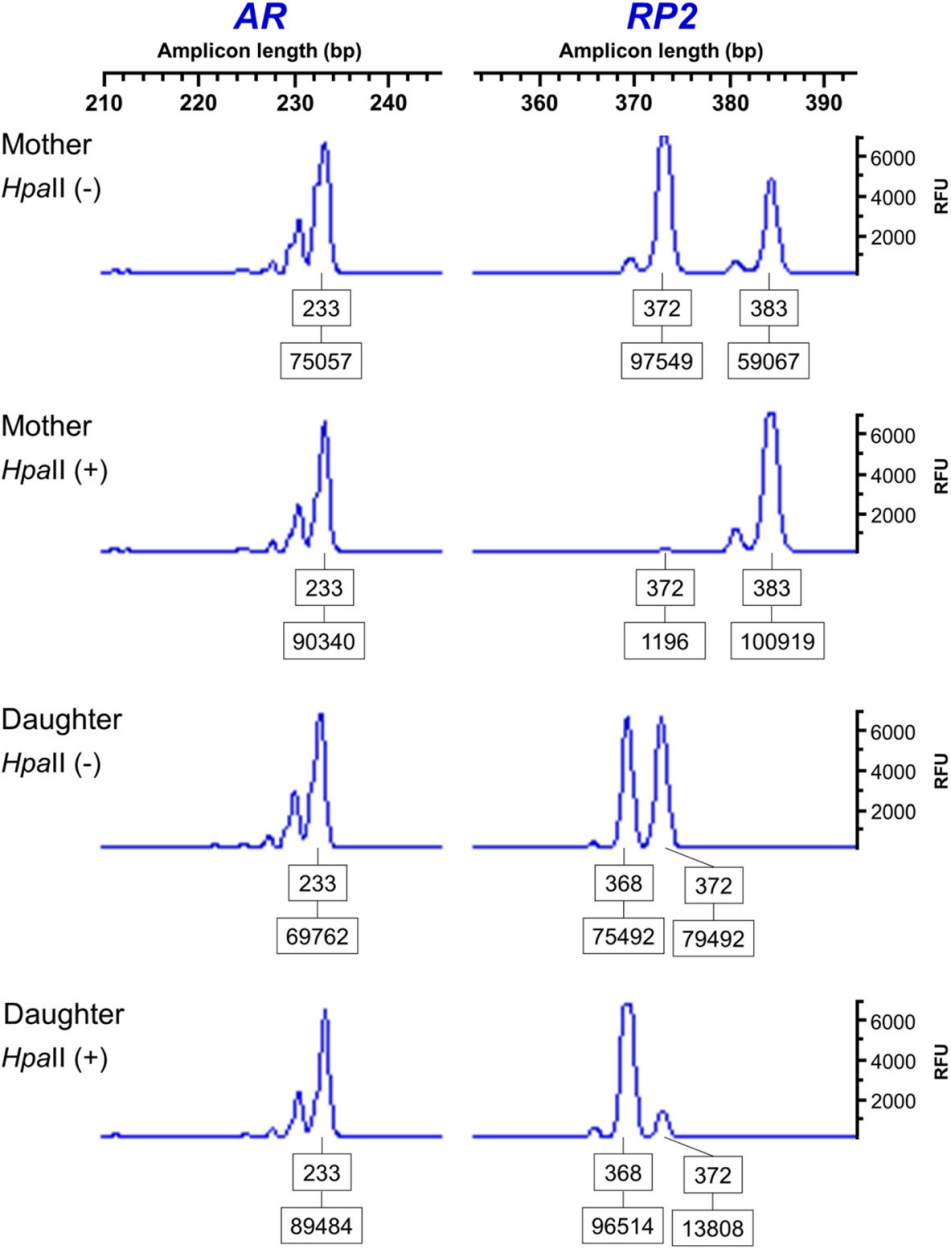
Results of X inactivation assay. Electropherograms using the 5meCpG-based RP2/AR repeat biplex PCR assay across the HpaII restriction site in the mother and daughter carrying a balanced X- autosometranslocation. Both mother and daughter are homozygous for the AR marker (allele 233bp). For the informative RP2 marker, the (X;22) translocated chromosome was identified by the maternal segregation pattern of the shared 372bp allele. The pattern of inactivation demonstrates that translocated (X;22) chromosome is active in 99.3% of the blood cells in the mother and in 88.0% of blood cells in the daughter. The boxed numbers correspond to the amplicon length (top) and the areas under the allele peaks (bottom). RFU: Relative fluorescence units

Both mother (C1) and sister (A2) were homozygous for the *AR* marker. Thus, this marker was uninformative in the XCI assay. Segregation analysis of the *RP2* alleles indicated that the X chromosome represented by the 372 bp allele corresponds to the X;22 translocated chromosome, and that it was preferentially active in both mother and daughter (Fig. 3). We estimated that the translocated chromosome was active in 99.3 % of the mother’s cells and in 88.0 % of the sister’s.

## Discussion

Molecular cytogenetic investigation has confirmed that the translocation present in the proband and his sister was maternally inherited. The coincident and unexpected finding of mosaicism of X-chromosome in the aunt (C3) and grandmother (G1) is intriguing. Chromosomal mosaicisms are not heritable since they always occur as post-zygotic segregation anomalies. Incidences of mosaicism occur as genetically altered clonal lineages that can arise following mitotic errors in normal cells. In addition there are also cases where a new lineage emerged from multiple trisomic or monosomic rescue events in altered cells [14–17]. The possibility of independent rescue events occurring in a single individual lineage is supported by the high chromosomal instability detected in studies of human embryogenesis [18–20].

There are a limited number of cases of reported mosaicism in which the clonally aneusomic line was inherited [21]. However, there are currently no reports of recurrence of identical mosaicism between members of the same family. In both aunt (C2) and grandmother (G1), it is likely that the mosaic karyotype with normal, trisomic and monosomic lineages for the X chromosome arose by chromosomal non-disjunction from a chromosomally normal zygote. It is unlikely that the postulated occasional post-zygotic error, occurred by coincidence in both mother and daughter. But it is possible that, at least in the aunt (C2), one of the X aneuploid lineage was inherited. Hultén et al. [22] showed that most normal female fetuses are mosaics for trisomy 21 in their ovarian tissues. Considering that normal females can present detectable rates of aneuploid gametes we can expect a higher rate in mosaicism carriers. The detection of one 48, XXXX cell suggests that rescue events in a triple X cell have prompted the emergence of a 46, XX cell line and a 48, XXXX cell line. In this case, the normal strain would be strongly selected and the altered strain would be repressed.

The translocation (X;22) detected in the mother’s proband (C1) could be originated by an abnormal gamete from the grandmother (G1). Robles et al. [23] show that the pairing between trisomic chromosomes is slower than among disomic ones. Chromosome 22 is the second smallest autosomal chromosome and, on average, it binds to its homologous chromosome by only one or two chiasmata during meiosis. Thus, unlike the larger chromosomes, the loss of a single chiasma between chromosomes 22 is likely to cause errors on chromosome pairing and migration during meiosis [24]. Therefore, it is likely that the incorrect pairing between chromosomes 22 in grandmother gamete induced a pairing error with a single X chromosome, or an extra, unpaired on a trivalent form, followed by non-homologous recombination giving rise to the translocation.

We propose that the chromosomal mosaicism detected in the grandmother gave rise to the different chromosome abnormalities present in the next two generations of this family. This suggests that carriers of X-chromosome aneuploidy mosaicism may, in some cases, be related to segregation errors in the oogenesis. The recurrent miscarriages of the aunt would also corroborate this hypothesis.

Array-CGH results showed no genomic gains or losses in the translocation breakpoint regions or anywhere in the genome of the infertile patients and their family members. To this extent, on this resolution level, the translocation appears to be balanced. We suggest that the phenotype of male infertility occurred by disruption of spermatogenesis resulting from pairing and chromosome segregation error due to the translocation.

Another possible explanation for male infertility in our patients could be spreading of X chromosome inactivation into the active chromatin at the translocated region of chromosome 22. Previous studies have shown that such autosomal translocation may lead to association with the sex body at pachytene of meiosis, leading to gradual heterochromatinization that could affect transcription of necessary genes for the meiotic process leading to cell death and male infertility [25]. It is possible that, in our patients, the translocated 22 chromosome fragment was silenced during meiosis. Future studies using techniques such late-replication analysis would be required to elucidate this hypothesis.

We also cannot totally exclude the possibility the translocations may have caused a position effect by disrupting regulatory regions of genes related to the phenotype. Our findings corroborate the literature where is verified no preference for the involvement of a specific chromosome in the X-autosomal translocations and shows that infertility is inevitable for male carriers regardless of the position of the X chromosome breakpoint [26].

Additionally, although the X-chromosome inactivation assay for both mother and sister revealed a nonrandom X inactivation, they showed different percentages. While the mother presented a percentage of 99.3 % of activity of the translocated X-chromosome, the sister presented 88.0 %. Classical studies demonstrated that the X-autosome balanced translocation remains active and approximately 99 % of the non-translocated chromosome X is inactive, probably due to a survival disadvantage [27]. Wolff et al. [28] studied the X inactivation pattern in individuals with structurally abnormal X chromosome and verified that patients without complete skewing of X inactivation (>97 %) presented different phenotypic abnormalities. The sister’s proband had an inactivation of approximately 12 % of the translocated chromosome (X;22) and, consequently, the inactivation of this derivative X chromosome could be spreading to the translocated fragment of the chromosome 22 [29, 30]. Considering that mother and daughter presented no genomic gains or losses and were considered as carriers of truly balanced translocations, we could associate the differences between the X-chromosome inactivation patterns with the phenotypic expression.

## Conclusions

This was the first report so far of a reciprocal translocation involving a sex chromosome that was probably originated by errors in oogenesis as well as a case of recurrent mosaicism in the same family. Also, we verified a possible correlation between the phenotype of infertility and the pattern of X-chromosome inactivation in a female carrier of X;22 translocation.

## Consent

Written informed consent was obtained from the patients for publication of this Case report. A copy of the written consent is available for review by the Editor-in-Chief of this journal.

## Abbreviations

array-CGH: Microarray-based comparative genomic hybridisation
CNV: Copy number variation
FISH: Fluorescent in situ hybridization
QF-PCR: Quantitative fluorescence polymerase chain reaction
XCI: X-chromosome inactivation

## References

[1] De Braekeleer M, Dao TN (1991). Cytogenetic studies in male infertility: a review. Hum Reprod.

[2] Egozcue S, Blanco J, Vendrell JM, García F, Veiga A, Aran B, Barri PN, Vidal F, Egozcue J. Human male infertility: chromosome anomalies, meiotic disorders, abnormal spermatozoa and recurrent abortion. Hum Reprod Update. 2000;6(1):93–105. 10.1093/humupd/6.1.93 10711834

[3] Suzuki T, Tsurusaki Y, Nakashima M, Miyake N, Saitsu H, Takeda S, Matsumoto N (2014). Precise detection of chromosomal translocation or inversion breakpoints by whole-genome sequencing. J Hum Genet.

[4] Gajecka M, Gentles AJ, Tsai A, Chitayat D, Mackay KL, Glotzbach CD, Lieber MR, Shaffer LG. Unexpected complexity at breakpoint junctions in phenotypically normal individuals and mechanisms involved in generating balanced translocations t (1;22) (p36; q13). Genome Res. 2008;18(11):1733–42. 10.1101/gr.077453.108 PMC2577863 18765821

[5] Martin RH (2008). Cytogenetic determinants of male fertility. Hum Reprod Update.

[6] Dong Y, Du RC, Jiang YT, Wu J, Li LL, Liu RZ (2012). Impact of chromosomal translocations on male infertility, semen quality, testicular volume and reproductive hormone levels. J Int Med Res.

[7] Van Assche E, Bonduelle M, Tournaye H, Joris H, Verheyen G, Devroey P, Van Steirteghem A, Liebaers I. Cytogenetics of infertile men. Hum Reprod. 1996;11(4):1–26. 10.1093/humrep/11.suppl_4.1 9147109

[8] Delhanty JDA (2013). The origins of genetic variation between individual human oocytes and embryos: implications for infertility. Hum Fertil (Camb).

[9] Turner JMA (2007). Meiotic sex chromosome inactivation. Development.

[10] Gardner RJM, Sutherland GR, Shaffer LG (2011). Chromosome abnormalities and genetic counselin 4th ed.

[11] Zarrei M, MacDonald JR, Merico D, Scherer SW (2015). A copy number variation map of the human genome. Nat Rev Genet.

[12] Machado FB, Faria MA, Lovatel VL, da Silva AF A, Radic CP, De Brasi CD, Rios AF, de Sousa Lopes SM, da Silveira LS, Ruiz-Miranda CR (2014). 5meCpG Epigenetic Marks Neighboring a Primate-Conserved Core Promoter Short Tandem Repeat Indicate X-Chromosome Inactivation. PLoS One.

[13] Busque L, Paquette Y, Provost S, Roy DC, Levine RL, Mollica L, Gilliland DG. Skewing of X-inactivation ratios in blood cells of aging women is confirmed by independent methodologies. Blood. 2009;113(15):3472–4. 10.1182/blood-2008-12-195677 PMC4729536 19202126

[14] Kagami M, Kato F, Matsubara K, Sato T, Nishimura G, Ogata T (2012). Relative frequency of underlying genetic causes for the development of UPD (14) pat-like phenotype. Eur J Hum Genet.

[15] Kotzot D (2001). Complex and segmental uniparental disomy (UPD): review and lessons from rare chromosomal complements. J Med Genet.

[16] Shaffer LG, Agan N, Goldberg JD, Ledbetter DH, Longshore JW, Cassidy SB (2001). American College of Medical Genetics statement of diagnostic testing for uniparental disomy. Genet Med.

[17] Vetro A, Manolakos E, Petersen MB, Thomaidis L, Liehr T, Croci G, Franchi F, Marinelli M, Meneghelli E, Dal Bello B, et al. Unexpected results in the constitution of small supernumerary marker chromosomes. Eur J Med Genet. 2012;55(3):185–90. 10.1016/j.ejmg.2012.01.010 22342433

[18] Mantzouratou A, Delhanty JD (2011). Aneuploidy in the human cleavage stage embryo. Cytogenet Genome Res.

[19] Vanneste E, Voet T, Le Caignec C, Ampe M, Konings P, Melotte C, Debrock S, Amyere M, Vikkula M, Schuit F (2009). Chromosome instability is common in human cleavage-stage embryos. Nat Med.

[20] Voet T, Vanneste E, Van der Aa N, Melotte C, Jackmaert S, Vandendael T, Declercq M, Debrock S, Fryns JP, Moreau Y, et al. Breakage-fusion-bridge cycles leading to inv dup del occur in human cleavage stage embryos. Hum Mutat. 2011;32(7):783–93. 10.1002/humu.21502 21412953

[21] Robberecht C, Voet T, Utine GE, Schinzel A, de Leeuw N, Fryns JP, Vermeesch J. Meiotic errors followed by two parallel postzygotic trisomy rescue events are a frequent cause of constitutional segmental mosaicism. Mol Cytogenet. 2012;5(19). 10.1186/1755-8166-5-19 PMC3350457 22490612

[22] Hultén MA, Patel SD, Tankimanova M, Westgren M, Papadogiannakis N, Jonsson AM, Iwarsson E. On the origin of trisomy 21 Down syndrome. Mol Cytogenet. 2008;1(21). 10.1186/1755-8166-1-21 PMC2564957 18801168

[23] Robles P, Roig I, Garcia R, Ortega A, Egozcue J, Cabero LL, Garcia M. Pairing and synapsis in oocytes from female fetuses with euploid and aneuploid chromosome complements. Reproduction. 2007;133(5):899–907. 10.1530/REP-06-0243 17616720

[24] Hall HE, Surti U, Hoffner L, Shirley S, Feingold E, Hassold T (2007). The origin of trisomy 22: evidence for acrocentric chromosome-specific patterns of nondisjunction. Am J Med Genet A.

[25] Oliver-Bonet M, Ko E, Martin RH (2005). Male infertility in reciprocal translocation carries: the sex body affair. Cytogenet GenomeRes.

[26] Ma S, Yuen BH, Penaherrera M, Koehn D, Ness L, Robinson W (2003). ICSI and the transmission of X-autosomal translocation: a three-generation evaluation of X;20 translocation: case report. Hum Reprod.

[27] Mattei MG, Mattei JF, Ayme S, Giraud F (1982). X-autosome translocations: cytogenetic characteristics and their consequences. Hum Genet.

[28] Wolff DJ, Schwartz S, Carrel L (2000). Molecular determination of X inactivation pattern correlates with phenotype in women with a structurally abnormal X chromosome. Genet Med.

[29] Disteche CM (2012). Dosage Compensation of the Sex Chromosomes. Annu Rev Genet.

[30] Sharp AJ, Spotswood HT, Robinson DO, Turner BM, Jacobs PA (2002). Molecular and cytogenetic analysis of the spreading of X inactivation in X; autosome translocations. Hum Mol Genet.

