# Erratum to: Familial chromosomal translocation X; 22 associated with infertility and recurrent X mosaicism

**DOI:** 10.1186/s13039-016-0262-8

**Published:** 2016-07-05

**Authors:** Juliana Dourado Grzesiuk, Ciro Silveira Pereira, Carlos Henrique Paiva Grangeiro, Clarissa Gondim Picanço-Albuquerque, Flávia Gaona Oliveira-Gennaro, Filipe Brum Machado, Enrique Medina-Acosta, Ester Silveira Ramos, Maisa Yoshimoto, Lucia Martelli

**Affiliations:** Genetics Department, Ribeirão Preto Medical School, University of Sao Paulo, Ribeirao Preto, 14049-900 Brazil; Center of Biotecnology and Cellular Therapy, San Raphael Hospital, Salvador, 41253-190 Brazil; Center of Biosciences and Biotechnology, Darcy Ribeiro State University of Northern of Rio de Janeiro, Campos dos Goytacazes, 28013-600 Brazil; Department of Medical Genetics, Faculty of Medical and Dentistry, University of Alberta, Edmonton, Canada

## Erratum

Unfortunately, the original version of this article [1] contained an error. Figure 2a was missing.

**Fig. 2:**
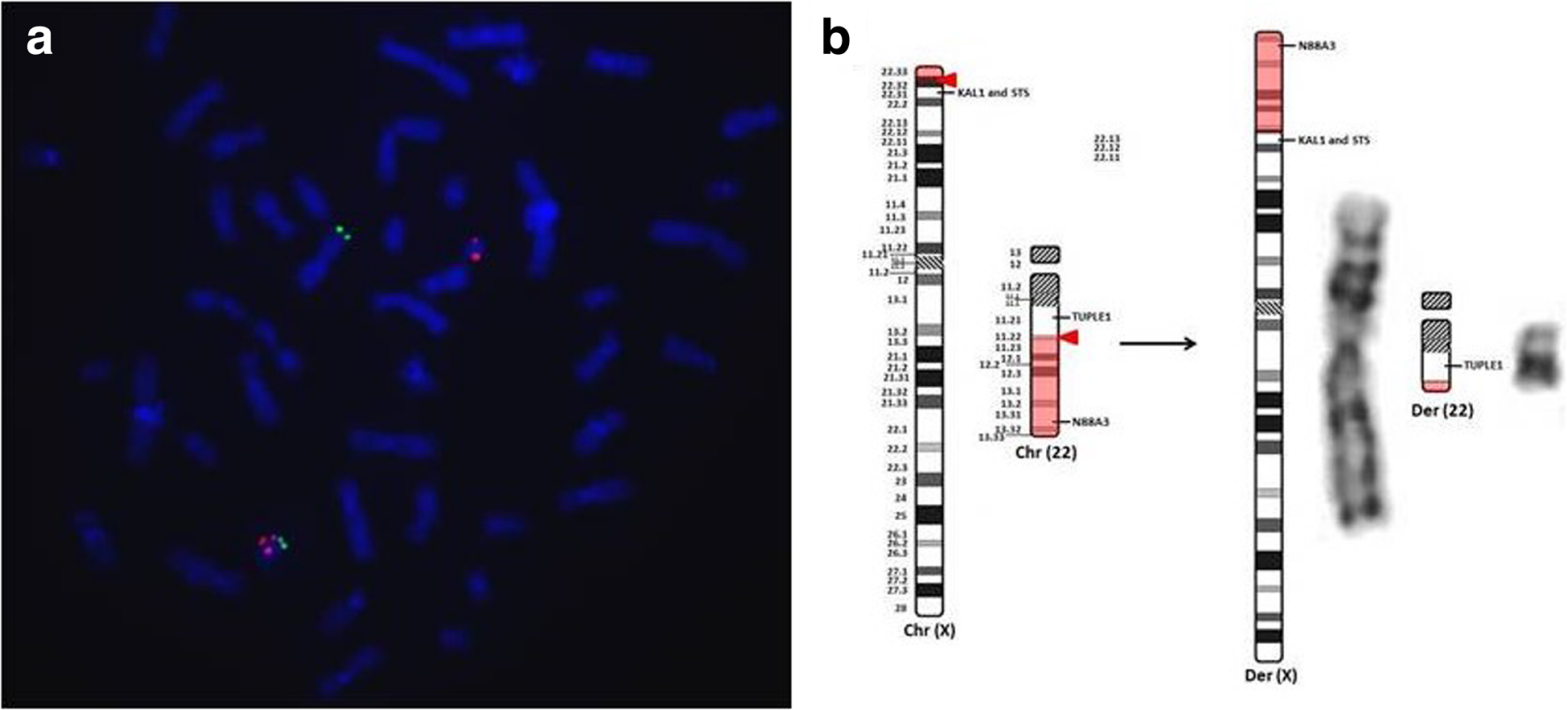
FISH and Cytogenetic analyses of the translocation cariers. **a** FISH technique from patient A2 showing the subtelomeric probe of chromosome 22 (*in green*) in the distal portion of the short arm of the translocated X chromosome, while probe for TUPLE1 gene is located at band q11.21 (*in red*), remaining on the der(22). **b** On the left side the ideograms from normal chromosomes X and 22 show the breakpoints (*red arrow*) and the translocated segments (*shaded in red*). On the right side are the schematic ideograms and the GTG banding pattern of the derivative chromosomes

The correct version of Fig. 2a,b can be found below. Figure 2 has been corrected in the original article [1] and is also included correctly below.
